# The effects of sodium butyrate supplementation on the expression levels of PGC-1α, PPARα, and UCP-1 genes, serum level of GLP-1, metabolic parameters, and anthropometric indices in obese individuals on weight loss diet: a study protocol for a triple-blind, randomized, placebo-controlled clinical trial

**DOI:** 10.1186/s13063-022-06891-9

**Published:** 2023-08-01

**Authors:** Parichehr Amiri, Seyed Ahmad Hosseini, Neda Roshanravan, Maryam Saghafi-Asl, Mitra Tootoonchian

**Affiliations:** 1 Student Research Committee, Ahvaz Jundishapur University of Medical Sciences, Ahvaz, Iran; 2 Nutrition and Metabolic Diseases Research Center, Clinical Research Institute, Ahvaz Jundishapur University of Medical Sciences, Ahvaz, Iran; 3 Department of Nutrition, School of Allied Medical Sciences, Ahvaz Jundishapur University of Medical Sciences, P.O. Box 61357-15794, Ahvaz, Iran; 4 Cardiovascular Research Center, Tabriz University of Medical Sciences, Tabriz, Iran; 5 Nutrition Research Center, Department of Clinical Nutrition, School of Nutrition and Food Sciences, Tabriz University of Medical Sciences, Tabriz, Iran; 6 Endocrine Research Center, Tabriz University of Medical Sciences, Tabriz, Iran

**Keywords:** Sodium butyrate, Obesity, Energy metabolism genes, Appetite, Randomized controlled trial

## Abstract

**Background:**

*Obesity* is a multifaceted disease characterized by an abnormal accumulation of adipose tissue. Growing evidence has proposed microbiota-derived metabolites as a potential factor in the pathophysiology of obesity and related metabolic conditions over the last decade. As one of the essential metabolites, butyrate affects several host cellular mechanisms related to appetite sensations and weight control. However, the effects of butyrate on obesity in humans have yet to be studied. Thus, the present study was aimed to evaluate the effects of sodium butyrate (SB) supplementation on the expression levels of peroxisome proliferator activated-receptor (PPAR) gamma coactivator-1α (PGC-1α), PPARα and uncoupling protein 1 (UCP1) genes, serum level of glucagon-like peptide (GLP1), and metabolic parameters, as well as anthropometric indices in obese individuals on a weight loss diet.

**Methods:**

This triple-blind randomized controlled trial (RCT) will include 50 eligible obese subjects aged between 18 and 60 years. Participants will be randomly assigned into two groups: 8 weeks of SB (600 mg/day) + hypo-caloric diet or placebo (600 mg/day) + hypo-caloric diet. At weeks 0 and 8, distinct objectives will be pursued: (1) PGC-1α, PPARα, and UCP1 genes expression will be evaluated by real-time polymerase chain reaction; (2) biochemical parameters will be assayed using enzymatic methods; and (3) insulin and GLP1 serum level will be assessed by enzyme-linked immunosorbent assay kit.

**Discussion:**

New evidence from this trial may help fill the knowledge gap in this realm and facilitate multi-center clinical trials with a substantially larger sample size.

**Trial registration:**

Iranian Registry of Clinical Trials: IRCT20190303042905N2. Registered on 31 January 2021.

## Introduction

Obesity is caused by a positive energy imbalance when energy consumed exceeds energy expenditure [1]. Obesity plays a crucial role in the genesis of many chronic disorders, including cardiovascular disease, type 2 diabetes mellitus (T2DM), rheumatoid arthritis, and various cancers, causing complications such as glucose intolerance, insulin resistance (IR), systemic inflammation, hyperlipidemia, and hypertension [2, 3]. The World Health Organization (WHO) estimates that the global prevalence of obesity has tripled since 1975 [4]. If current trends continue, by 2025, there will be 2.7 billion overweight people and more than 1 billion obese people [4]. This demonstrates that obesity has steadily increased over the last few decades and is now a global health concern [1].

Obesity is a multifactorial disease in which, in addition to heredity, environmental, social, physiological, and metabolic factors all play an essential role in its occurrence [5]. The appetite of obese people usually increases, while their energy expenditure decreases, owing to insufficient physical activity and brown adipose tissue (BAT) dysfunction [6].

BAT contributes significantly to energy expenditure by burning triglycerides (TG) and glucose in humans [7]. In recent years, researches on the relation of intestinal microbiota with obesity and epigenetic modifications have remarkably grown. The metabolic functions of the intestinal microbiota are the fermentation of dietary fiber and the production of short-chain fatty acids (SCFAs) [8]. In the lumen of the colon, the significant SCFAs are acetate (C2), propionate (C3), and butyrate (C4) in a 1:1:3 molar ratios. Increased bacterial fermentation of SCFAs contributes to the regulation of systemic energy by reducing hepatic production of glucose and lipids [9, 10].

Animal models of metabolic diseases have reported that among all SCFAs, butyrate has the highest anti-obesity activity [11]. There are several mechanisms through which butyrate works, including preventing weight gain caused by a high-fat diet (HFD), reducing serum TG, total cholesterol, glucose, IR, and improving hepatic steatosis [11, 12]. Firmicutes and Bacteroides are the two main types of butyrate-producing bacteria; previous research has shown that these bacteria are reduced in the obese people [13]. On the other hand, a study showed that the butyrate-producing bacteria are higher in lean people, and when these bacteria are transferred to the intestines of people with metabolic syndrome (MetS), their IR status improves [14].

Treatment of obesity and obesity-related disturbances depends mainly on diet, exercise, and drugs to treat specific components, such as orlistat, metformin, and statins [15, 16]. This pharmaceutical strategy can lead to drug interactions, as people with MetS may require several medications [17]. Additionally, treating one component of MetS can harm another [15]. In this regard, butyrate, a microbial metabolite, has recently sparked much interest as a safe supplement that could decrease weight, serum glucose, cholesterol, and blood pressure while having minimal side effects [11, 12, 18]. Butyrate, a histone deacetylase inhibitor, could hyper-acetylate transcription factors and change several genes expression level especially peroxisome proliferator activated-receptor (PPAR) gamma coactivator-1α (PGC-1α), PPARα, and uncoupling protein 1 (UCP1) [19, 20]. Furthermore, butyrate stimulates the secretion of some gastrointestinal anorexic hormones, including the glucagon-like peptide (GLP-1) [21].

Overall, evidence from in vivo and in vitro studies showed that butyrate has a remarkable impact on metabolic regulation and body composition [11]. Butyrate has been strongly linked to increased energy expenditure, fatty acid oxidation, and decreased energy intake. Therefore, it appears that butyrate can be considered a potential therapeutic agent for obesity and MetS. Hence, due to eminent anti-obesity effects of butyrate as well as lack of human clinical trials to evaluate the effects of butyrate in obesity, the present study was aimed to investigate the effects of sodium butyrate (SB) supplementation on the expression levels of PGC-1α, PPARα, and UCP1 genes, serum level of GLP-1, metabolic parameters, and anthropometric indices in obese individuals.

## Methods

### Study design and setting

A triple-blind, parallel-group, single-center and superiority placebo-controlled clinical trial with 1:1 allocation ratio will be conducted. The proposed RCT will take place in the Faculty of Nutrition and Food Sciences, Tabriz University of Medical Sciences, for 8 weeks to see how daily 600 mg SB supplementation affects obese people.

**Fig. 1:**
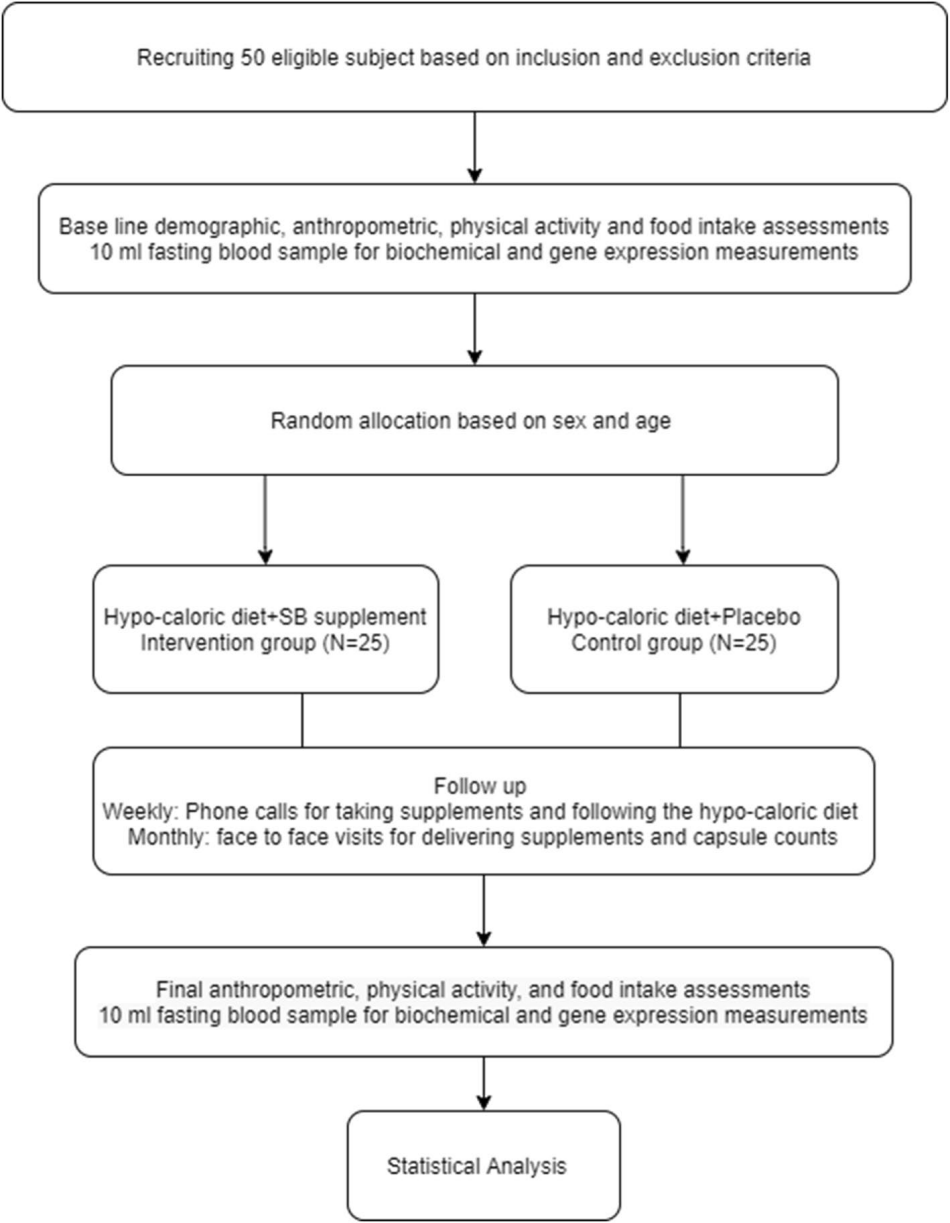
Study flow chart

Individuals will be recruited for clinical trial through widely distributed printed and social media advertisements from the health centers of Tabriz University of Medical Sciences (including hospitals, outpatient receptions, communities, and health clinics). Initial screening and enrollment will be done by a nutritionist. Screening will continue until the target sample size is achieved. Interpretation of laboratory results, analysis of body composition, and dietary intakes will be performed for individuals without charge as incentives. Figure 1 depicts the study flow chart.

### Participants

The study will involve 50 obese individuals. Participants will be both male and female aged 18 to 60 years old, with body mass index (BMI) of 30 to 40 kg/m^2^; non-inclusion criteria will be included: lack of interest in continuing the study; having diabetes, kidney diseases, heart failure, rheumatic diseases, cancers, liver failure, gastrointestinal disorders; history of obesity surgery; following a weight loss diet or taking any supplements or weight loss drugs in the last 6 months; smoking; and pregnancy, lactation, or menopause.

### Registries and ethics

Eligible participants will receive the study protocol and will then be able to have an informed discussion with the participating consultant. Trained researcher will obtain written consent from patients willing to participate in the trial. Consent forms will be provided for all parents involved in the trial. A material consent form will be taken for extra biological samples (serum, plasma, and stool) which will be used in future studies. If the data collection in the ancillary study is not included in the informed consent process of main RCT, participants should provide written consent.

This protocol was approved by Ethics Committee of Ahvaz Jundishapur University of Medical Sciences (approval number: IR.AJUMS.REC.1399.845). The research was also registered at the Iranian Registry of Clinical Trials (IRCT20190303042905N2). The protocol must be amended if any changes can affect the conduct of the study, the participant benefits, or supplement use safety, including a change in study objectives, study design, procedures, sample size, or significant administrative changes. It will be agreed upon by IRCT and approved by the Ethics Committee before implementation. The participant’s information will be kept in the lockers in restricted areas. Data collection forms and biological samples will be given a unique code in order to ensure confidentiality.

### Sample size

**Fig. 2:**
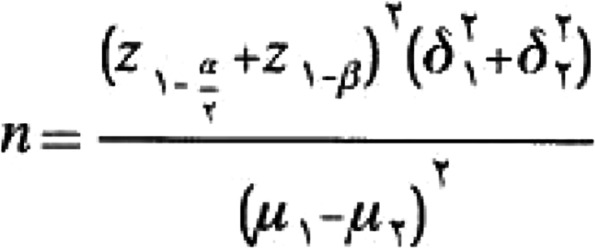
Sample size calculating formula. *α* is the significance level, *β* is the power of the test, *δ* is standard deviation, and μ1_μ2 is difference between the means

The sample size was estimated based on serum changes of GLP-1 [22], 95% confidence interval, and 80% power (two-sided *α* = 0.05 and *β* = 0.2) using sample size formula (Fig. 2) suggested for comparison of mean between the two groups when the endpoint is quantitative data in parallel designed RCTs. Furthermore, the means and standard deviation (SD) [23] of GLP-1 levels in the study mentioned above were as follows: Δμ = 17.84; SD1 = 17.93; and SD2 = 22.49. Lastly, considering a 20% dropout rate, 25 subjects were calculated for each group. Individuals who fulfilled the eligibility criteria will be included in the RCT.

### Blinding and randomization of participants

All participants will be randomly assigned into two groups (intervention or placebo), stratified by age (two age groups: 18–39 years and 40–60 years) and gender (male and female). The intervention group will receive SB supplementation + hypo-caloric diet (*n* = 25), and the placebo group will receive placebo capsule + hypo-caloric diet (*n* = 25). The random block design will be performed by a third person using the Random Allocation Software (RAS). After the baseline evaluations, and checking the inclusion and exclusion criteria by two of the main researchers, eligible person will be allocated into the intervention or control groups using sealed envelopes.

In case of emergency, where it is essential to be aware of the treatment allocation. The clinical consultant who is not involved in the enrolment and intervention process will have access to the unblinding envelopes provided for every individual. Further evaluation and required medical intervention will be done depend on situation. The reason of emergency unblinding will be recorded in the case report form and revealed treatment code will not be recorded in the study documents or verbally disclosed to the researchers or participants.

Random numbers will be labeled as SB or placebo bottles, and even or odd numbers will be randomly assigned to the study groups. To maintain blindness, instead of A or B, the third person will use specific codes. The bottles will be similar in appearance and weight to achieve blinding. The treatment allocation will be concealed from participants, researchers, and outcome assessors.

### Intervention

The intervention group will receive one SB capsule )600 mg( with breakfast for 60 days. The dosage of SB supplementation was determined, based on the previous study [22]. SB supplement (550 mg butyrate, 50 mg hydroxypropyl methylcellulose, medium-chain triglycerides (MCT), sodium hydroxide, and purified water) will be provided by Body Bio Company (Body Bio, USA). The control group will receive the same amount of placebo capsules for 60 days. Pharmacy Faculty of Tabriz University of Medical Sciences will provide placebo capsules (Tabriz, Iran, containing 600 mg of carboxymethyl cellulose). Size and color of SB capsules and placebo capsules are exactly similar. Both groups will receive SB and placebo capsules every 30 days. The remaining capsules returned by participants will be counted to determine adherence to the supplements in all patients. Adherence will be defined as consuming 90% of the supplements.

**Fig. 3:**
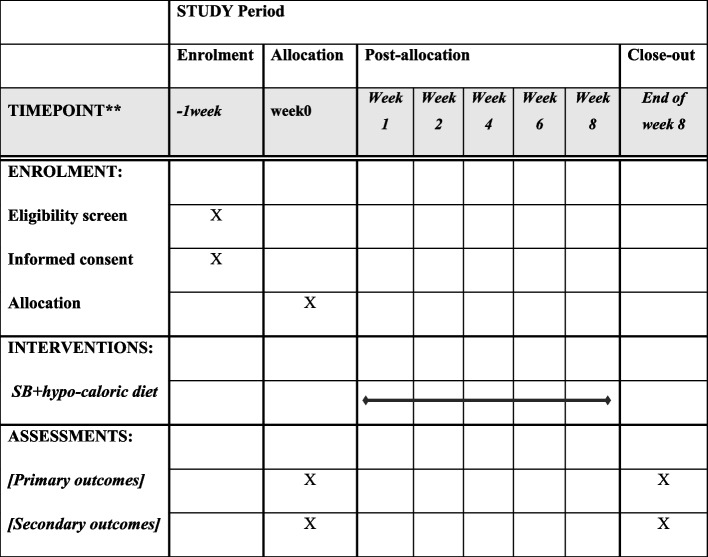
SPIRIT figure: schedule for enrollment, intervention, and assessment

An expert dietician will design an individualized hypo-caloric diet for each participant. The Mifflin St. Jeor equation will be used to calculate total energy expenditure requirements [24]. Then, 500 kcals of the estimated energy needs will be deducted. The hypo-caloric diet composition will consist of 10–15% protein, 25–30% fat, and 55–60% carbohydrate. Compliance with diet will be checked weekly by phone calls as well as a 3-day food record at the onset and end-point of the study. Figure 3 depicts the schedule for the present study.

The research team will provide weekly communications via phone calls and messages to inform the participants about obesity, the current status of the study, and plans for the next phase, emphasizing study benefits as well as to acknowledge their support. In week 4, there will be a face-to-face session with the participant to assess anthropometric changes and help to maintain interest in study.

### Criteria for discontinuing or modifying allocated intervention

Since SB may affect the composition of the gut microbiota, temporary gastrointestinal symptoms such as bloating or diarrhea may occur. Taking the recommended daily dose regularly, gastrointestinal tolerance improves, and the symptoms usually disappear. However, the trial medication should be withdrawn from subjects reporting gastro-intestinal intolerance. This should be reported as an adverse event.

### Evaluation of diet, physical activity, and appetite

A checklist will be filled out at baseline, which includes demographic data and medical and drug history. For evaluation of dietary intake, the participants will be asked to keep a 3-day (one weekend and two weekdays) food record. Energy and nutrients analysis will be performed using Nutritionist IV (Hearst Corporation, USA). Physical activity level (PAL) will be calculated by the International Physical Activity Questionnaire short form (IPAQ-SF) [25]. To assess appetite, the participants will complete a visual analog scale (VAS) at baseline and end of study [26].

### Anthropometric assessments

Body weight will be measured for subjects wearing minimal clothing (Seca scale) and height will be measured by a wall-mounted meter. To calculate BMI, weight (kg) will be divided by height squared (m^2^). An accurate measurement of waist circumference (WC) will be taken, using a tape meter at the midpoint between the lowest rib and iliac crest at the end of normal expiration. Hip circumference (HC) will be measured at its widest point over the greatest trochanters. Waist/hip ratio (WHR) will also be calculated. Fat-free mass (FFM), FFM percent, fat-mass (FM), and FM percent, as well as visceral fat, will be estimated through MC-780 TANITA body composition analyzer (Tanita, Amsterdam, The Netherlands).

### Biochemical assays

Five milliliters of venous blood sample will be taken after a 12-h overnight fasting in vacuumed gel separator tubes at baseline and end of the study. Blood samples will be centrifuged at 3000 RPM for 5 min to extract the serum. Serum aliquots will be frozen (− 80 °C) to measure GLP1 (μg/mL) and insulin (μU/mL) using enzyme-linked immunosorbent assay (ELISA) kits.

Fasting blood sugar (FBS) (mg/dL), triglycerides (TG), total cholesterol (TC), high-density lipoprotein cholesterol (HDL-C), aspartate transaminase (AST), alanine transferase (ALT), and alkaline phosphatase (ALP) will be measured by enzymatic colorimetric kits. The Friedewald equation will be used to compute low-density lipoprotein cholesterol (LDL-C) concentrations. Calculation of the homeostatic model assessment for IR (HOMA-IR) will be done using the following formula: HOMA-IR = [fasting insulin (μIU/mL) × fasting glucose (mg/dL)]/405 [27].

### Gene expression assay

Five milliliters of blood samples will be collected in EDTA/K2 tubes. Peripheral blood mononuclear cells (PBMCs) will be isolated using Ficoll-Hypaque density gradient centrifugation. Total RNA extraction will be done using Trizol reagent, according the manufacturer’s instruction. After designing primers, the mRNA gene expression of PGC-1α, PPARα, and UCP1 genes will be applied using real-time polymerase chain reaction (real time-PCR). In real time-PCR tests, the housekeeping gene will be β-actin. The fold change of gene expressions will be calculated based on the Livak formula (2^–ΔΔCt^) [28].

### Outcomes

Primary outcomes will be changes in means of anthropometrics indices, serum levels of GLP1, and fold change of PGC-1α, PPARα, and UCP1 genes between the control and intervention groups. Secondary outcomes are to compare the changes in means of lipid profile (serum levels of TG, TC, HDL-C, and LDL-C), FBS, fasting plasma insulin level, HOMA-IR, ALT, AST, and ALP between the control and intervention groups. All primary and secondary outcomes will be evaluated at the baseline and the end of the study (day 60 after intervention) for all participants. All outcomes and time points are specified in Fig. 3.

### Statistical analysis

Data analysis of the present RCT will be conducted on an intention to treat (ITT) participants. ITT means outcome data obtained from all participants are included in the data analysis, regardless of protocol adherence. The data will be checked for accuracy and completeness at random intervals. The data will be expressed as means ± SD. For each variable, the percentage change will be calculated, as follows: [(E–B)/B ×100]; *E* is the end value, and *B* is the baseline value of the variable.

The Kolmogorov-Smirnov test will be used to examine the normality of the data. The intervention arm (SB) will be compared against the control (placebo) for all primary and secondary outcomes. The paired sample *t*-test and independent sample *t*-test will be performed for the comparison of parametric variables within and between groups, respectively. For non-parametric variables, the Mann-Whitney test and Wilcoxon signed rank test will be applied. Confounding factors including baseline values will be controlled using the analysis of covariance (ANCOVA) test. IBM SPSS software (version 21) (Armonk, NY, USA) will be used to analyze the data. A *P*-value less than 0.05 will be considered a statistically significant.

### Adverse effects, safety, and data monitoring

SB supplementation at dosage of 600 mg/day is not known to have any side effect [11]. Two supervisors from a Data Monitoring Committee (DMC) will monitor the outcome of the RCT. The DMC is independent of the study organizers (sponsor and trial investigators).

Furthermore, the report of potential side effects will also be forwarded to the Ethics Committee of Ahvaz University of Medical Sciences.

## Discussion

Recent studies have revealed that microbiota-associated metabolites may play an imperative role in obesity and metabolic disturbances related to it [11, 12]. Butyrate action is linked to the activation of mitochondria and energy expenditure enhancement via activation of PGC-1α. PGC-1α is a transcriptional activator identified as a viable molecular target for dietary interventions [29]. PGC-1α regulates energy metabolism and fatty acid oxidation by expressing mitochondrial biogenesis and respiration genes by interacting with several transcription factors such as PPARα and UCP1 [30]. PPARα is also expressed in various tissues involved in fat oxidation in the heart, vascular, and endothelial cells and regulates the expression of genes involved in the transport and beta-oxidation of fatty acids in the mitochondria [31]. PPARα stimulates lipolysis and increases the uptake of fatty acids by increasing the expression of fat transporters and lipid metabolizing enzymes. Studies have also shown that the activated PPARα can improve glucose metabolism and weaken inflammatory processes [32, 33].

Uncoupling proteins (UCPs) are protein transporters in the mitochondrial membrane which separate the oxidative metabolism pathway from adenosine diphosphate (ADP) phosphorylation and adenosine triphosphate (ATP) synthesis via directing protons to the mitochondrial matrix to produce heat [34]. Therefore, UCPs are candidate genes for obesity and metabolic complications associated with obesity, and measuring the expression of UCPs is a method for assessing the oxidation of fatty acids [33, 35, 36]. On the other hand, activating genes related to UCPs have been identified as BAT activation markers. Increasing BAT activity is also vital for energy metabolism and is considered as a key factor that affects insulin sensitivity [37].

Based on several health benefits of Butyrate and the lack of adequate human studies, RCTs are required to evaluate the effects of SB supplementation on markers of metabolic pathway and anthropometric indices in the obese subjects. The strengths of this triple-blind randomized controlled clinical trial are the evaluation of the effects of SB supplementation and individualized hypo-caloric diet plan on obese subjects. We expect that the outcomes of this trial, whether positive or negative, will fill the gap in the literature and facilitate the development of multi-center clinical trials with a substantially larger sample size.

## Trial status

Recruitment for this trial has begun at June 2021 and is expected to be completed by November 2021 (now, it is underway).

## Data Availability

The datasets will be accessible to all principal investigators. Any participant identifying information will be removed from data dispersed to the research team to ensure confidentiality. The dataset generated during the current study will be available via the corresponding author upon reasonable request. The results and findings of the study will be released through publications in scientific literature and conference presentations. All those who involved in the design and implementation of this protocol will be author in the articles resulting from this research project.

## Abbreviations

BAT: Brown adipose tissue
BMI: Body mass index
ELISA: Enzyme-linked immunosorbent assay
GLP1: Glucagon-like peptide 1
HDL-C: High-density lipoprotein cholesterol
HFD: High-fat diet
HOMA-IR: Homeostasis model assessment of insulin resistance
ITT: Intention to treat
IR: Insulin resistance
MetS: Metabolic syndrome
LDL-C: Low-density lipoprotein cholesterol
PCR: Polymerase chain reaction
PPAR: Peroxisome proliferator activated-receptor
PGC-1α: Peroxisome proliferator activated receptor gamma coactivator-1α
RCT: Randomized controlled trial
SB: Sodium butyrate
SCFA: Short-chain fatty acid
SD: Standard deviation
T2DM: Type 2 diabetes mellitus
TC: Total cholesterol
TG: Triglycerides
UCP1: Uncoupling protein 1
WC: Waist circumference
WHR: Waist-to-hip ratio
